# Influence of Season and Soil Properties on Fungal Communities of Neighboring Climax Forests (*Carpinus cordata* and *Fraxinus rhynchophylla*)

**DOI:** 10.3389/fmicb.2020.572706

**Published:** 2020-10-28

**Authors:** Ki Hyeong Park, Seung-Yoon Oh, Shinnam Yoo, Jonathan J. Fong, Chang Sun Kim, Jong Won Jo, Young Woon Lim

**Affiliations:** ^1^School of Biological Sciences and Institute of Microbiology, Seoul National University, Seoul, South Korea; ^2^Department of Biology and Chemistry, Changwon National University, Changwon, South Korea; ^3^Science Unit, Lingnan University, Tuen Mun, Hong Kong; ^4^Forest Biodiversity Division, Korea National Arboretum, Pocheon, South Korea

**Keywords:** arbuscular mycorrhizal fungi, ectomycorrhizal fungi, temperate forest, plant-soil interaction, soil fungal community

## Abstract

Trees in forest ecosystems constantly interact with the soil fungal community, and this interaction plays a key role in nutrient cycling. The diversity of soil fungal communities is affected by both environmental factors and host tree species. We investigated the influence of both of these factors by examining the total fungal communities in the rhizospheric soil of climax tree species that have similar ecological roles (*Carpinus cordata*, an ectomycorrhizal [ECM] tree, and *Fraxinus rhynchophylla*, an arbuscular mycorrhizal [AM] tree) in temperate forests with continental climates of Mt. Jeombong, South Korea. Fungal communities were assessed by Illumina-MiSeq sequencing the internal transcribed spacer (ITS) region of environmental DNA, and comparing their environmental factors (season and soil properties). We found that soil fungi of the two forest types differed in terms of community structure and ecological guild composition. The total fungal community composition changed significantly with seasons and soil properties in the *F. rhynchophylla* forest, but not in the *C. cordata* forest. However, potassium and carbon were significantly correlated with fungal diversity in both forests, and a positive correlation was found only between symbiotrophs of *C. cordata* and the carbon to nitrogen (C/N) ratio. Thus, the effects of environmental factors on soil fungal communities depended on the host trees, but some factors were common in both forests. Our results indicate that individual tree species should be considered when anticipating how the fungal communities will respond to environmental change.

## Introduction

Fungi in forests play key roles in plant diversity and productivity (Van Der Heijden et al., 2008). There is a wide diversity of associations between fungi and plants categorized by how a fungus gets organic matter from plants – symbiotrophic, saprotrophic, and pathotrophic. Among the symbiotrophic fungi, mycorrhizal fungi receive photosynthetic products from plant in exchange for mediating environmental stresses, increasing foraging area, and enhancing water and nutrient absorption through a hyphal network with plants (Smith and Read, 2010; Kramer et al., 2012). Most terrestrial plants require at least one type of mycorrhizal association to properly grow and reproduce (Brundrett and Tedersoo, 2018). Saprotrophic fungi decompose organic matter and are involved in carbon cycling and nutrient mobilization in forests (Kramer et al., 2012; van der Wal et al., 2013), while pathotrophic fungi retrieve nutrients by harming living plants and can control plant populations (Nguyen et al., 2016). Each trophic mode can be divided to several guilds according to their ecological lifestyle (Tedersoo et al., 2014).

Plants can also influence fungal communities by modifying microhabitats with leaf fall and litter (Aponte et al., 2010). The soils surrounding plants have different properties due to decomposition of organic matter, accumulation of carbon, and microbial residues such as amino sugar (Soudzilovskaia et al., 2015; Lin et al., 2017; Craig et al., 2018). This phenomenon results in different carbon and nitrogen cycling rates between host tree species, such as slower carbon and nitrogen cycling in ectomycorrhizal (ECM) forests than in arbuscular mycorrhizal (AM) forests (Phillips et al., 2013; Averill et al., 2014; Sulman et al., 2017). Trees can be divided into four types based on mycorrhizal associations, and the most abundant types being AM and ECM trees (Brundrett, 2009). AM trees include species in the genera *Acer* and *Ulmus* and family Podocarpaceae and have symbiotic relationships with fungal species in Glomeromycota (Brundrett, 2009; Helgason and Fitter, 2009). ECM trees include species in the families, such as Betulaceae, Fagaceae, and Pinaceae, and are associated with fungi in Ascomycota and Basidiomycota (Brundrett, 2009; Tedersoo et al., 2010). ECM forests are known to store more carbon and have a higher carbon to nitrogen (C/N) ratio than AM forests (Vesterdal et al., 2013; Averill et al., 2014). However, it should be noted that plants with dual mycorrhization of AM and ECM have been reported (Brundrett, 2017; Tedersoo and Brundrett, 2017).

The heartleaf hornbeam (*Carpinus cordata*) is an ECM deciduous tree native to northeast Asia that grows in shaded, moist valley forests (Smith and Read, 2010; Kwon et al., 2014). *Fraxinus rhynchophylla*, a species of ash tree distributed in East Asia, grows well on nutrient-rich soils and is an AM tree (Ambriz et al., 2010). Both *C. cordata* and *F. rhynchophylla* are climax species commonly found in Korean temperate forests (Lee et al., 1990; Cho et al., 2005; Park et al., 2016). Previous studies have helped us begin to understand the relationship between fungi and species of *Carpinus* and *Fraxinus*. For instance, study on ECM fungi associated with *Fagus*, *Tilia*, and *Carpinus* trees suggested the host preference of ECM fungi, as more than half of species were found in one host (Lang et al., 2011). Meanwhile, the influence of abiotic factors on fungal communities were found in pure *F. mandshurica* forest, where higher relative abundance of saprotophic fungal were found in soil with higher carbon and nitrogen compared to that of mixed forest of *P. koraiensis* and *F. mandshurica* (Wu et al., 2019).

*Carpinus cordata* and *F. rhynchophylla* climax forests are both found in well-protected valleys in Korea (Cho et al., 2005; Park et al., 2016). This characteristic provides a unique opportunity to compare fungal communities across soil properties and tree types (ECM vs. AM trees), while controlling for environmental variation because the forests are located so close to each other. This study is part of a larger project by the Korea National Arboretum to understand the interactions between fungi and major tree species in Korea. Various features of the fungal community (alpha diversity, community structure, and ecological guild composition) from the two forest types were compared to determine seasonal variation and the influence of soil properties. We aimed to elucidate the influence of host tree type, in particular AM and ECM trees, on soil fungal communities, while also investigating the effects of season and soil properties (pH, carbon, nitrogen, phosphorus, potassium, and water content).

## Materials and Methods

### Study Site, Soil Collection, and Chemical Analyses

The study was carried out in temperate forests of Mt. Jeombong, Seoraksan National Park, Inje County, Gangwon Province, South Korea (38.032124, 128.463275, altitude 780–830 m). Sampling was permitted by the Seoraksan National Park Authority. We choose one *C. cordata* forest and one *F.* rhy*nchophylla* forest that were adjacent (within 350 m) to minimize the effects of environmental variation. Several shrubs belonging to Ericaceae were found in each sampling site, but forests were composed of single tree species in each stand. This approach allowed us to focus on seasonal variation and the influence of soil properties on the fungal communities. We sampled soil from three individual trees from each forest for each of the four seasons in 2018; selected trees were visually inspected to have no disease symptoms and were at least 20 m away from other sampled trees. Rhizospheric soil samples were collected in triplicate for each tree, from 0 to 10 cm soil layer that was close to root of the host tree, after removing surface litter. In total, 36 soil samples were collected from each forest type (three trees × three replicates × four seasons). Soil samples were placed on ice while being transported to the laboratory. Upon arrival, triplicate soil samples from the same tree were mixed and sieved to 2.0 mm (= 12 sample × 2 forest types = 24 total samples). Half of each Mixed soil sample was sent to National Instrumentation Center for Environmental Management (Seoul National University, Seoul, South Korea) for soil property analysis on the same day of collection. The following soil properties were measured: pH, total organic carbon (TOC), total nitrogen (TN), NH_4_^+^, total phosphorus (TP), water content, total potassium (TK), and C/N ratio (based on TOC/TN). The remaining half of each sample was stored at −80°C until DNA extraction.

### DNA Extraction, Amplification, and Sequencing

DNA was extracted from each of the 72 soil samples (two forest types × three trees × three replicates of soil mixture × four seasons) within a week of sampling. Extraction was performed from 0.25 g of soil sample with the Power-Soil® DNA Isolation Kit according to the manufacturer’s instructions (MoBio Laboratories, Carlsbad, CA). After extraction, the internal transcribed spacer 2 (ITS2) region was amplified using the primers ITS3 and ITS4 (White et al., 1990) attached with Illumina sequencing adaptors. To minimize the effects of PCR bias, PCR was conducted three times for each DNA sample using an AccuPower PCR PreMix Kit (Bioneer, Deajeon, South Korea). We used the following PCR conditions: 94°C for 5 min; 30 cycles of 94°C for 30 s, 55°C for 30 s, and 72°C for 40 s; and a final extension of 72°C for 10 min. The size of PCR products was visually checked on 1% agarose gel (BIOFACT, Daejeon, South Korea). Triplicate PCR products for a sample were pooled and purified using an Expin™ PCR SV Kit (GeneAll Biotechnology, Seoul, South Korea). To attach multiple index delimiters (MID), we followed the Nextera XT Index Kit protocol (Illumina, San Diego, CA, USA). After purification, DNA quality and concentration from PCR products were quantified with NanoDrop 2000 (Thermo Fisher Scientific, Waltham, MA, USA) and pooled at equimolar concentrations before sequencing. The 72 samples were sequenced by 300 bp × 2 paired-end sequencing on an Illumina MiSeq platform (Macrogen, Seoul, South Korea). Raw sequence data were deposited on NCBI Sequence Read Archive (SRA) under Project ID PRJNA638267.

### Bioinformatics and Statistical Analyses

All sequence analyses were performed using the Quantitative Insights Into Microbial Ecology (QIIME) v.1.8.0. pipeline (Caporaso et al., 2010). We used fastq-join to merge paired-end sequences and filter out low-quality sequences (Q < 20, length < 200 bp). operational taxonomic units (OTUs) were clustered based on a 97% similarity threshold using an average linkage method on USEARCH v. 5.2.236 (Edgar, 2010). For taxonomic assignments, the most abundant sequence was selected as the representative sequence of an OTU. Reference sequences from UNITE v. 7.2 (Kõljalg et al., 2013) and Seoul National University Fungal Collection (SFC) were used for taxonomic assignment, following the criteria of Tedersoo et al. (2014). Chimeric sequences were filtered against the reference database with UCHIME (Edgar et al., 2011). Singleton OTUs and non-fungal sequences were removed and all samples were rarified to the minimum number of sequences (30,000 reads) and pooled (90,000 reads) before further analysis. Fungal functional guilds were assigned according to the FUNGuild database (Nguyen et al., 2016). The relative abundance of each OTU was calculated as the ratio of the number of sequences reads of an OTU per total sequence reads in a batch of samples.

Four alpha diversity indices (Chao1 richness, Shannon’s diversity and equitability, and Good’s coverage) were calculated in QIIME and displayed with R software (R Core Team, 2019). A Kruskal-Wallis test was performed to identify the statistically significant relationships between the alpha diversity indices and season with Fisher’s least significant difference (LSD) test as a *post hoc* test adjusted by the Bonferroni method using the agricolae package (de Mendiburu and de Mendiburu, 2019). Variation in fungal communities among soil samples was visualized by a principal coordinate analysis (PCoA) based on Bray-Curtis dissimilarity index using the phyloseq package (McMurdie and Holmes, 2013). Vectors of soil property factors were fit with the envfit function in the vegan package. To determine the significance of the variations, differences in fungal community compositions across seasons and host trees were evaluated using permutational multivariate analysis of variance (PERMANOVA) with 999 permutations, implemented as “adonis” in the vegan package (Oksanen et al., 2018). Spearman correlation analyses were used to examine the correlation between alpha diversity indices, fungal guilds (in relative abundance), and abiotic factors. The correlation coefficient and value of *p* were calculated with the ggcorrplot package in R (Kassambara, 2016).

## Results

### Fungal Composition and Guild Classification

A total of 8,740,440 sequence reads and 17,008 OTUs were initially obtained from the 72 samples. After filtering, 8,301,778 sequence reads and 10,856 OTUs remained. For individual samples, 32,789–202,543 sequence reads were obtained. The rarefaction curves of the observed number of OTUs for each sample were sufficiently saturated, with more than 98.5% Good’s coverage (data not shown). After rarifying the data to 30,000 reads/samples, we obtained 9,580 OTUs. Sequence data of three replicates from the same tree and the same season were combined into a single sample (90,000 reads). The average length of sequences was 389 ± 55 bp. We analyzed data from a total of 24 samples (two forest types × three trees × four seasons).

**Figure 1:**
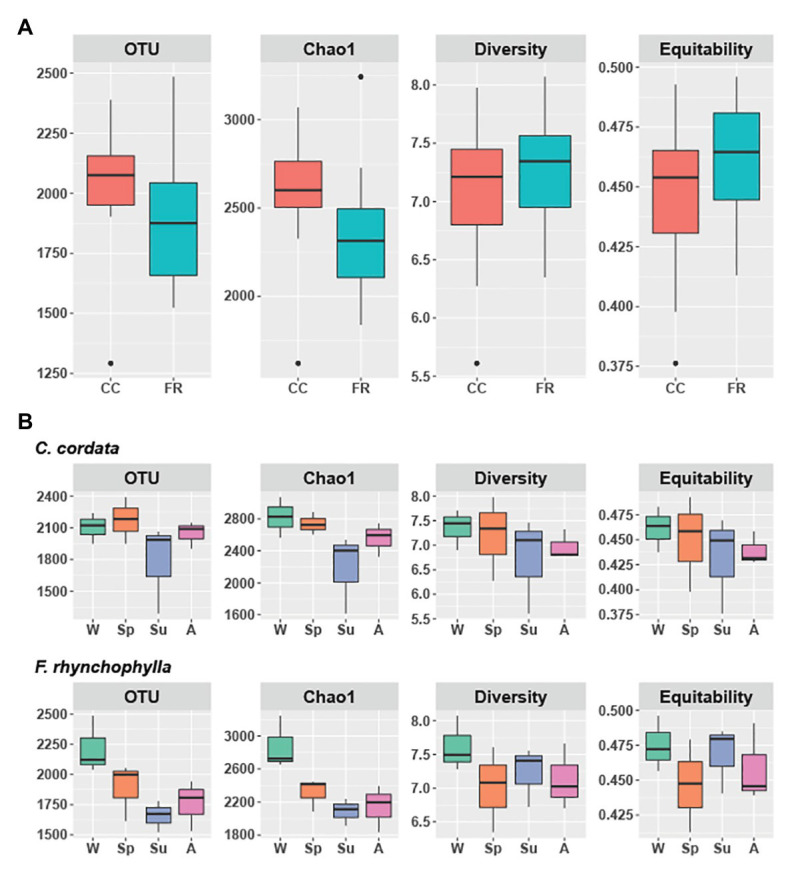
Alpha diversity of soil fungal communities from *Carpinus cordata* and *Fraxinus rhynchophylla* forests. **(A)** Overall alpha diversity of the rhizospheric soil under the dominant tree species in each forest and **(B)** Seasonal variation in alpha diversity in *C. cordata* and *F. rhynchophylla*: W (winter), Sp (spring), Su (summer), and A (autumn). Richness is represented by the number of OTUs and Chao1. Diversity and equitability were estimated with Shannon’s index. Significant differences in alpha diversity indices were tested using Kruskall-Wallis test with LSD Fisher *post hoc* test adjusted by the Bonferroni method. The box indicates the first and third quartiles, and black line in the middle indicates the median value.

**Figure 2:**
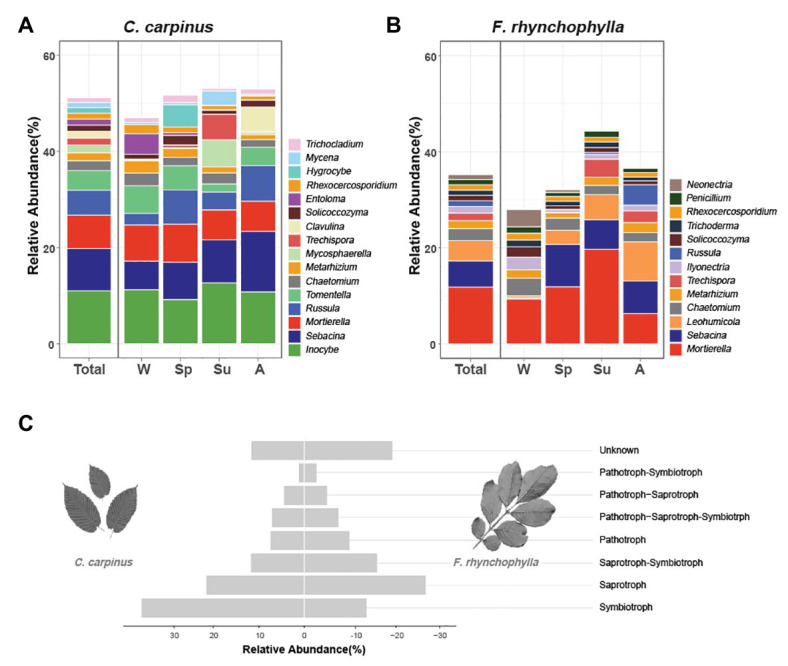
Taxonomic distribution of soil fungal communities in *Carpinus cordata* and *Fraxinus rhynchophylla* forests. The average relative abundances of genera in soil fungal communities of **(A)** *C. cordata* and **(B)** *F. rhynchophylla* forests are represented according to seasonal change. The genera with relative abundances less than 1% are not shown. **(C)** Average relative abundance of taxa belonging to each trophic mode in fungal communities of *C. cordata* and *F. rhynchophylla* forests. Unknown = taxonomic groups with unassigned trophic modes.

In the *C. cordata* forest, a total of 6,549 OTUs were obtained, and 1,292–2,389 OTUs were found from each sample. (Figure 1A). Basidiomycota was the most abundant (46.0%), followed by Ascomycota (45.9%) and Mortierellomycota (7.1%). At the genus level, *Inocybe*, *Sebacina*, *Mortierella*, *Russula*, and *Tomentella* were the most abundant (Figure 2A). The relative abundance of sequence reads belonging to genus *Inocybe* (9.2–12.7%) and *Mortierella* (6.3–7.5%) were mostly stable across seasons, while that of *Sebacina* was higher in autumn (12.6%) than in other seasons (5.9–8.9%). The relative abundance of *Russula* was lower in summer and winter (2.2–3.7%) than in spring and autumn (7.1–7.4%), while that of *Tomentella* was higher in spring and winter (5.0–5.7%) than in summer (1.7%). For trophic modes, symbiotrophs were the most abundant, followed by saprotrophs and saprotroph-symbiotrophs (Figure 2C). Among the symbiotrophs, an average of 67.9% of OTUs were EM and 0.4% were AM. Among the major genera, *Inocybe*, *Sebacina*, *Russula*, and *Tomentella* were classified as symbiotrophs. Saprotrophs were represented by *Trechispora* and *Trichocladium*. *Mortierella* was categorized as a saprotroph-symbiotroph. Several pathotrophic fungi were also found (Table 1).

**Table 1:** Average relative abundance, trophic mode, and guild of major genera (>1%) in the fungal communities of rhizospheric soil in *Carpinus cordata* and *Fraxinus rhynchophylla* forests.

Tree	Genus	Relative abundance (%)	Trophic mode^*^	Guild
*Carpinus cordata*	*Inocybe*	10.981	Sym	Ectomycorrhizal
	*Sebacina*	8.792	Sym	Ectomycorrhizal-orchid mycorrhizal-root associated biotroph
	*Mortierella*	6.992	Sap-Sym	Endophyte-litter saprotroph-soil saprotroph-undefined saprotroph
	*Russula*	5.176	Sym	Ectomycorrhizal
	*Tomentella*	4.056	Sym	Ectomycorrhizal
	*Chaetomium*	2.082	Pat-Sap-Sym	Animal pathogen-dung saprotroph-endophyte-epiphyte-plant saprotroph-wood saprotroph
	*Metarhizium*	1.654	Pat	Animal pathogen
	*Mycosphaerella*	1.581	Pat	Plant pathogen
	*Trechispora*	1.502	Sap	Wood saprotroph
	*Clavulina*	1.379	Sym	Ectomycorrhizal
	*Solicoccozyma*	1.303	Unk	Unknown
	*Entoloma*	1.253	Pat-Sap-Sym	Ectomycorrhizal-fungal parasite-soil saprotroph-undefined saprotroph
	*Rhexocercosporidium*	1.212	Pat	Plant pathogen
	*Hygrocybe*	1.158	Sap-Sym	Undefined saprotroph-undefined biotroph
	*Mycena*	1.063	Pat-Sap	Leaf saprotroph-plant pathogen-undefined saprotroph-wood saprotroph
	*Trichocladium*	1.022	Sap	Undefined saprotroph
*Fraxinus rhynchophylla*	*Mortierella*	11.832	Sap-Sym	Endophyte-litter saprotroph-soil saprotroph-undefined saprotroph
	*Sebacina*	5.471	Sym	Ectomycorrhizal-orchid mycorrhizal-root associated biotroph
	*Leohumicola*	4.193	Sap	Undefined saprotroph
	*Chaetomium*	2.525	Pat-Sap-Sym	Animal pathogen-dung saprotroph-endophyte-epiphyte-plant saprotroph-wood saprotroph
	*Metarhizium*	1.641	Pat	Animal pathogen
	*Trechispora*	1.577	Sap	Wood saprotroph
	*Ilyonectria*	1.344	Sap	Undefined saprotroph
	*Russula*	1.199	Sym	Ectomycorrhizal
	*Solicoccozyma*	1.169	Unk	Unknown
	*Trichoderma*	1.085	Sap	Undefined saprotroph
	*Rhexocercosporidium*	1.085	Pat	Plant pathogen
	*Penicillium*	1.068	Sap	Undefined saprotroph
	*Neonectria*	1.061	Pat	Plant pathogen

* Sap, saprotroph; Sym, symbiotroph; Pat, pathotroph; Unk, unknown.

Trophic modes and guilds were assigned according to FUNGuild database (Nguyen et al., 2016).

In the *F. rhynchophylla* forest, a total of 7,196 OTUs were obtained, and 1,524–2,485 OTUs were found in each sample (Figure 1A). Ascomycota was the most abundant (56.7%), followed by Basidiomycota (29.9%) and Mortierellomycota (12%). At the genus level, *Mortierella*, *Sebacina*, *Leohumicola*, *Chaetomium*, and *Metarhizium* were the most abundant (Figure 2B). The abundances of *Chaetomium* (1.9–3.7%) and *Metarhizium* (1.1–2.1%) were stable throughout the year. *Sebacina* and *Leohumicola* had the lowest relative abundance in winter (0.2 and 0.5%, respectively), and the abundance of *Mortierella* was the lowest in autumn (6.3%). For trophic modes, saprotrophs were the most abundant and included genera *Leohumicola* and *Trechispora*. The next most abundant trophic modes were saprotroph-symbiotrophs and symbiotrophs (Figure 2C). The average abundance of EM among the symbiotrophs was 40.6% and that of AM was 1.0%. Among the major genera, the proportion of saprotrophic fungi in *F. rhynchophylla* forest (*Mortierella*, *Leohumicola*, *Chaetomium*, *Trechispora*, *Ilyonectria*, *Trichoderma*, and *Penicillium*) was higher than that of *C. cordata* forest (Table 1).

**Figure 3:**
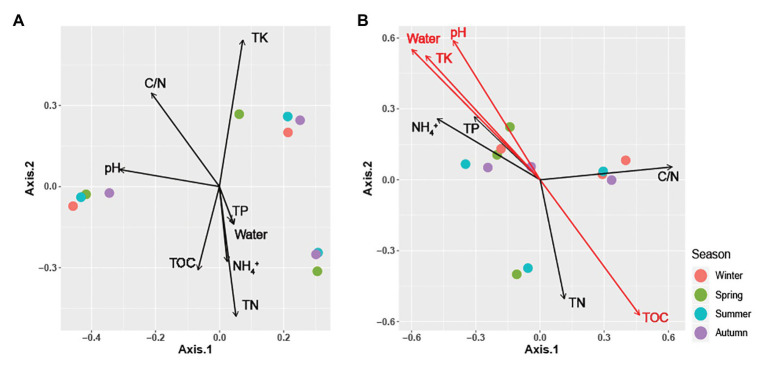
Principal coordinate analysis (PCoA) plots using Bray-Curtis distances matrices of **(A)** *Carpinus cordata* and **(B)** *Fraxinus rhynchophylla* soil fungal communities. Dot colors represent seasons: purple (Winter), green (Spring), blue (Summer), and red (Autumn). Soil parameters with significant relationships (*p* < 0.05) are indicated by red arrows.

Although the differences between respective alpha diversity indices were non-significant between the two forest types, the Chao1 richness and number of OTUs were higher in *C. cordata* (*p* = 0.07 for Chao1 and *p* = 0.08 for OTUs; Figure 1A). The seasonal differences in alpha diversity indices are shown in Figure 1B. The number of OTUs was the lowest in summer in both forest types. OTU and Chao1 indices in *F. rhynchophyla* forest showed a marginal association among seasons (*p* = 0.07 for OTUs and *p* = 0.06 for Chao1). The number of OTUs was slightly lower in summer than winter (*p* = 0.07), while Chao1 was different between autumn and winter (*p* = 0.07) in the *post hoc* test. No significant seasonal differences were detected in the *C. cordata* community diversity, but the *F. rhynchophylla* community showed a significant change in diversity across seasons (Figure 3, Supplementary Table 1). The compositions of fungal communities were significantly different between tree species (*R*^2^ = 0.254, *p* < 0.001; Figure 2).

### Correlation Between Soil Properties and Fungal Community

**Figure 4:**
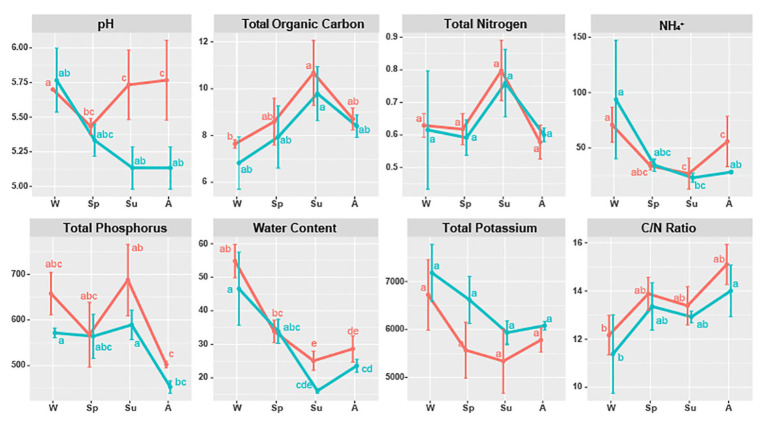
Soil properties of *Carpinus cordata* (red) and *Fraxinus rhynchophylla* (blue) forests separated by seasons. Measured soil properties are pH, TOC (Total Organic Carbon, %), TN (Total Nitrogen, %), NH_4_^+^ (ammonium ion, mg/kg), TP (Total Phosphorus, mg/kg), water (Water Content, %), TK (Total Potassium, mg/kg), and C/N ratio (carbon to nitrogen ratio, TOC/TN). Letters (a–e) were used as labels to indicate significant differences between means of each soil property in Kruskal-Wallis test with Bonferroni correction.

All soil properties except pH showed similar seasonal patterns between forest types (Figure 4). In the PERMANOVA analysis of the *C. cordata* forest, soil properties were not related to fungal communities (Figure 3A, Supplementary Table 1). However, pH, TOC, water content, TK, and C/N ratio had significant effects on fungal communities of *F. rhynchophylla*, while TN, NH_4_^+^, and TP had non-significant effects (Figure 3B, Supplementary Table 1). Results from the environmental fitness analysis mostly corroborated the PERMANOVA results in both forest types; the one exception was the effect of the C/N ratio on fungal communities, which was not significant in *F. rhynchophylla* (*p* = 0.109) for the environmental fitness analysis, but was significant for PERMANOVA analysis. Similarly, the correlation between soil properties and alpha diversity indices was different between forest types (Table 2). For *C. cordata*, water content and TK showed a significantly positive correlation with the number of OTUs and Chao1 richness index, while TOC showed a significantly negative correlation with the Chao1 richness index (Table 2A). In *F. rhynchophylla*, a significant positive correlation was detected between TK and four alpha diversity indices (OTU, Chao1, diversity, and equitability), and there were negative correlations among TOC, TN, and Chao1 index (Table 2B). For trophic modes, C/N ratio showed a negative correlation with the relative abundance of pathotroph-saprotroph-symbiotroph group in both forest types. In *C. cordata*, the relative abundance of symbiotroph group showed a positive correlation with the C/N ratio. In contrast, the relative abundance of saprotroph-symbiotroph group in *F. rhynchophylla* showed a positive correlation with TOC and TN, while the relative abundance of pathotroph-symbiotroph group showed a negative correlation with TOC and a positive correlation with TK.

**Table 2:** The result of Spearman correlation coefficient test between alpha diversity indices, relative abundance of trophic modes and soil properties in (A) *Carpinus cordata* and (B) *Fraxinus rhynchophylla* forests.

**A**									
		**pH**	**TOC**	**TN**	**NH_4_**	**TP**	**Water**	**TK**	**C/N**
Alpha diversity	Number of OTU	−0.147	−0.538	−0.28	0.371	0.035	0.648^*^	0.72^*^	−0.245
	Chao1	−0.111	−0.594^*^	−0.266	0.273	0.203	0.634^*^	0.832^*^	−0.364
	Diversity	−0.011	0.007	0.007	0.021	0.070	0.224	0.49^*^	−0.014
	Equitability	−0.011	0.077	0.063	−0.084	0.126	0.091	0.420	−0.084
Trophic Mode (relative abundance)	Sym	0.132	0.462	−0.161	−0.063	−0.238	−0.119	0.042	0.727^*^
	Sap	0.200	−0.364	0.224	−0.063	0.329	0.067	0.336	−0.664
	Sap-Sym	−0.429	−0.21	−0.294	0.021	−0.105	0.305	0.014	0.189
	Pat	0.347	−0.056	0.490	−0.154	0.497	−0.207	0.000	−0.643
	Pat-Sym	−0.261	−0.811	−0.629	0.490	−0.231	0.364	0.371	−0.196
	Pat-Sap	−0.197	−0.182	−0.028	−0.308	0.070	−0.165	0.273	−0.357
	Pat-Sap-Sym	0.143	−0.483	−0.014	0.399	0.371	0.298	−0.035	−0.545^*^
**B**									
		**pH**	**TOC**	**TN**	**NH_4_**	**TP**	**Water**	**TK**	**C/N**
Alpha diversity	Number of OTU	0.300	−0.34	−0.399^*^	0.308	−0.455	0.263	0.629^*^	−0.049
	Chao1	0.536	−0.62^*^	−0.608^*^	0.606	−0.231	0.585	0.692^*^	−0.245
	Diversity	0.427	−0.273	−0.175	0.256	−0.294	0.175	0.706^*^	−0.07
	Equitability	0.406	−0.287	−0.182	0.266	−0.287	0.182	0.741^*^	−0.084
Trophic Mode (relative abundance)	Sym	−0.423	0.025	−0.161	−0.179	−0.427	−0.2	−0.112	0.308
	Sap	0.250	−0.298	−0.182	0.186	−0.147	0.256	0.252	−0.224
	Sap-Sym	−0.215	0.417^*^	0.608^*^	−0.399	0.399	−0.469	−0.238	−0.175
	Pat	0.134	−0.179	0.077	0.025	0.049	0.053	0.028	−0.35
	Pat-Sym	0.490	−0.413^*^	−0.378	0.578	0.392	0.599	0.497^*^	−0.455
	Pat-Sap	−0.279	0.483	0.406	−0.41	−0.147	−0.431	−0.559	0.413
	Pat-Sap-Sym	0.511^*^	−0.375	0.014	0.525^*^	0.657	0.494^*^	0.280	−0.72^*^

* Indicate significant correlation (*p* < 0.05).

TOC, total organic carbon; TN, total nitrogen; NH_4_^+^, Ammonium ion; TP, total phosphorus; TK, total potassium; C/N, carbon to nitrogen ratio (TOC/TN); Sap, saprotroph; Sym, symbiotroph; Pat, pathotroph.

## Discussion

### Overall Diversity and Composition of Fungi Associated With ECM and AM Trees

The fungal community was significantly different between the two forest types. A high relative abundance of ECM fungi was found in the *C. cordata* forest, a result similar to those of previous studies on other *Carpinus* species (Tyler, 1992; Rudawska et al., 2019). Several ECM fungi were found in rhizopheric soil of *F. rhynchophylla* as well. Although *F. rhynchophylla* is known as an AM tree (Ambriz et al., 2010), it was also reported to have a symbiotic relationship with EM fungi (Teste et al., 2020). *Sebacina* and *Russula* were the major ECM fungi in both forest types. *Sebacina* was reported to be a major ECM fungus in oak forests of Mt. Jeombong (Oh et al., 2018) and is known to be abundant in late-stage forests rather than early-stage forests (Long et al., 2016). Likewise, *Russula* is dominant in the late successional stages of many forests, including those with *Carpinus* and *Fraxinus* species (Tedersoo et al., 2010; Tedersoo and Nara, 2010; Li et al., 2013). Mt. Jeombong is a well-preserved national park, so the high relative abundance of late successional ECM fungi in both forests reflects the climax forest stages. Other ECM fungi (*Clavulina*, *Inocybe*, and *Tomentella*), known as late-successional ECM fungi (Smith et al., 2002; Tedersoo and Nara, 2010; Lang et al., 2011; Kałucka and Jagodziński, 2017) were more abundant in the *C. cordata* forest. AM fungi were two times more abundant in the *F. rhynchophylla* forest than the *C. cordata* forest, although overall relative abundance of OTUs belonging to AM fungi in both forest types was low. The reason of low relative abundance of AM OTUs may be that we did not use primers specifically targeting AM fungi, and a complementary study with such primers is needed to complete our understanding of *F. rhynchophylla*’s fungal community. However, if presence of both AM and ECM is verified, as found in our study, this might reflect dual-mycorrhization of *F. rhynchophylla*, as reported in roots of *Fraxinus* species (Ambriz et al., 2010; Lindig-Cisneros et al., 2019; Teste et al., 2020).

The saprotrophic fungal composition showed the opposite pattern from that of the ECM composition. The relative abundance of saprotrophic taxa among sequence reads of total OTUs (*Mortierella*, *Leohumicola*, *Chaetomium*, and *Trechispora*) were higher in the *F. rhynchophylla* forest than the *C. cordata* forest. This result may be related to different litter decomposition rates of AM and ECM trees (Midgley et al., 2015). Soil and litter quality are known to affect the saprotrophic fungal community (Aponte et al., 2013), and the decomposition rate of litter is faster in AM than ECM trees (Phillips et al., 2013; Averill et al., 2014; Sulman et al., 2017). In addition, AM trees are likely to provide more litter biomass than ECM trees (Rosling et al., 2016). *Mortierella* was the major saprotrophic-symbiotrophic genus in both forest types. In association with plant roots, *Mortierella* is known to decompose dead fungal hyphae (Brabcová et al., 2016), promote plant growth, and suppress phytopathogenic nematodes (Eroshin and Dedyukhina, 2002; Al-Shammari et al., 2013). *Mortierella* was commonly reported in soil and roots samples from various environments (Summerbell, 2005; Curlevski et al., 2010). *Metarhizium* is a widespread entomopathogen found in soil and insects (Roberts and Leger, 2004). While its activity in soil is unclear, plant growth promotion by suppressing plant pathogenic fungi and entomopathogenic activities were reported (Leger, 2008). Other plant pathogens (*Mycosphaerella* and *Neonectria*) are also commonly found in diseased plant tissues or soil as an opportunistic pathogens (Zhang et al., 2005; Menkis and Burokienė, 2012).

### Effect of Season and Soil Properties on Soil Fungal Communities

While patterns of soil properties were similar in ECM (*C. cordata*) and AM (*F*. *rhynchophylla*) tree forests, fungal communities associated with *C. cordata* and *F. rhynchophylla* showed different responses to changes in season and soil properties. The fungal community associated with *F. rhynchophylla* showed temporal changes in relative abundance of major genera (Figure 2B). In contrast, no significant seasonal differences were detected in the fungal community associated with *C. cordata*. While the fungal communities in ECM and AM forests are generally affected by the season (Giachini et al., 2004; Bennett et al., 2013; Guadarrama et al., 2014; Voříšková et al., 2014; Santalahti et al., 2016), the opposite is also reported in ECM forests (Smith et al., 2007; Matsuoka et al., 2016). Priority effects – or the impact a species has on the community due to arriving first – might have caused a lack in seasonal shifts in *C. cordata*. If the early arriving fungi colonized root tips, later arriving fungi can be excluded from roots independent of environmental fluctuations (Dickie et al., 2012; Fukami, 2015). However, as patterns of fungal communities can change across years (Matsuoka et al., 2016), further research is needed to explain this phenomenon.

The relationship between the composition and abundance of a fungal community and soil properties was reported in previous studies, varying based on the locality and the characteristics of the sampling site (Yang et al., 2011; Voříšková et al., 2014; Žifčáková et al., 2016). In our study, the effect of soil properties on fungal community composition was generally significant in the *F. rhynchophylla* forest. Especially, we found that pH, potassium, and water content were positively correlated with each other, while TOC was negatively correlated in our result (Figure 3). However, their effect on alpha diversity indexes and trophic modes varied in *C. cordata* and *F. rhynchophylla* depending on soil properties (Table 2). Positive correlation between alpha diversity indices and TK were found in both forests, while richness and TOC showed negative correlations. These results indicate similarities among alpha diversity indices of fungal communities with different hosts, even though they had different compositions. There were also positive correlations with richness of soil fungal communities and TK contents in soil, while organic carbon was negatively linked to richness in both trees in both tree species. Potassium uptake is known to be improved by mycorrhization, and this improvement provide benefits to plants in the form of abiotic stress tolerance and phosphorus homeostasis maintenance (Dominguez-Nuñez et al., 2016). Meanwhile, a decrease of fungal biomass, diversity, and degrading activities were reported after carbon input by litter decomposition, but its mechanism is uncertain (Allison et al., 2007). In contrast, a positive correlation between C/N ratio and relative abundance of symbiotrophs was found only in *C. cordata*. High abundance of ECM in symbiotrophs of *C. cordata* might be associated with this result, as high C/N ratio was positively correlated with relative abundance of ECM taxa in previous studies (Chen et al., 2019), and ECM facilitate nitrogen uptake by releasing oxidative enzymes (Bödeker et al., 2014), but further investigation would develop our understanding of these processes.

## Conclusion

In this study, we uncovered differences in fungal diversity and soil properties between neighboring climax forests (*C. cordata* and *F. rhynchophylla*) using a metabarcoding approach. Although seasonal patterns of soil properties were similar across the two forest types, soil fungal communities differed based on the season and soil properties only in *F. rhynchophylla*. Our results suggest the importance of considering characteristics of host trees, as different climax forests may respond to changes (seasonal and soil properties) in different ways.

## Data Availability Statement

The datasets presented in this study can be found in online repositories. The names of the repository/repositories and accession number(s) can be found at: https://www.ncbi.nlm.nih.gov/bioproject/PRJNA638267/.

## Author Contributions

CK, JJ, and YL contributed to conceiving and designing the experiments. KP performed the experiments and analyzed the data with assistance from S-YO. KP and SY wrote the manuscript with revisions from JF, S-YO, and YL. All authors contributed to the article and approved the submitted version.

### Conflict of Interest

The authors declare that the research was conducted in the absence of any commercial or financial relationships that could be construed as a potential conflict of interest.
