# Supplementary material for: Influence of Season and Soil Properties on Fungal Communities of Neighboring Climax Forests (Carpinus cordata and Fraxinus rhynchophylla)
Source: Front Microbiol. 2020 Oct 28;11:572706. doi: 10.3389/fmicb.2020.572706 (PMC7655983; doi:10.3389/fmicb.2020.572706)
Supplement: Supplementary file 1 [file Table_1.docx]

Supplementary Material

# Supplementary Table 1. PERMANOVA analysis results based on Bray-Curtis dissimilarities using abundance data of fungal communities in relation to season and soil properties for 1.1) *Carpinus rhynchophylla* and 1.2) *Fraxinus rhynchophylla* dataset.

- 1. *Carpinus rhynchophylla*

|  |  | Df | SumsOfSqs | MeanSqs | F.Model | R^2^ | Pr(>F) |
| --- | --- | --- | --- | --- | --- | --- | --- |
| Season | Season | 3 | 0.47093 | 0.15698 | 0.48818 | 0.17302 | 0.968 |
|  | Residuals | 7 | 2.25087 | 0.32155 | 0.82698 |  |  |
|  | Total | 10 | 2.7218 | 1 |  |  |  |

|  |  | Df | SumsOfSqs | MeanSqs | F.Model | R^2^ | Pr(>F) |
| --- | --- | --- | --- | --- | --- | --- | --- |
| pH | pH | 1 | 0.2134 | 0.2134 | 0.76565 | 0.0784 | 0.637 |
|  | Residuals | 9 | 2.5084 | 0.27871 | 0.9216 |  |  |
|  | Total | 10 | 2.7218 | 1 |  |  |  |

|  |  | Df | SumsOfSqs | MeanSqs | F.Model | R^2^ | Pr(>F) |
| --- | --- | --- | --- | --- | --- | --- | --- |
| TOC | TOC | 1 | 0.15036 | 0.15036 | 0.52625 | 0.05524 | 0.899 |
|  | Residuals | 9 | 2.57145 | 0.28572 | 0.94476 |  |  |
|  | Total | 10 | 2.7218 | 1 |  |  |  |

|  |  | Df | SumsOfSqs | MeanSqs | F.Model | R^2^ | Pr(>F) |
| --- | --- | --- | --- | --- | --- | --- | --- |
| TN | TN | 1 | 0.21783 | 0.21784 | 0.78296 | 0.08003 | 0.573 |
|  | Residuals | 9 | 2.50397 | 0.27822 | 0.91997 |  |  |
|  | Total | 10 | 2.7218 | 1 |  |  |  |

|  |  | Df | SumsOfSqs | MeanSqs | F.Model | R^2^ | Pr(>F) |
| --- | --- | --- | --- | --- | --- | --- | --- |
| NH_4_^+^ | NH4 | 1 | 0.19066 | 0.19065 | 0.67791 | 0.07005 | 0.744 |
|  | Residuals | 9 | 2.53115 | 0.28124 | 0.92995 |  |  |
|  | Total | 10 | 2.7218 | 1 |  |  |  |

|  |  | Df | SumsOfSqs | MeanSqs | F.Model | R^2^ | Pr(>F) |
| --- | --- | --- | --- | --- | --- | --- | --- |
| TP | TP | 1 | 0.12889 | 0.12889 | 0.44738 | 0.04735 | 0.977 |
|  | Residuals | 9 | 2.59291 | 0.2881 | 0.95265 |  |  |
|  | Total | 10 | 2.7218 | 1 |  |  |  |

|  |  | Df | SumsOfSqs | MeanSqs | F.Model | R^2^ | Pr(>F) |
| --- | --- | --- | --- | --- | --- | --- | --- |
| Water | Water | 1 | 0.14934 | 0.14934 | 0.52247 | 0.05487 | 0.892 |
|  | Residuals | 9 | 2.57247 | 0.28583 | 0.94513 |  |  |
|  | Total | 10 | 2.7218 | 1 |  |  |  |

|  |  | Df | SumsOfSqs | MeanSqs | F.Model | R^2^ | Pr(>F) |
| --- | --- | --- | --- | --- | --- | --- | --- |
| TK | TK | 1 | 0.23075 | 0.23075 | 0.8337 | 0.08478 | 0.548 |
|  | Residuals | 9 | 2.49105 | 0.27678 | 0.91522 |  |  |
|  | Total | 10 | 2.7218 | 1 |  |  |  |

|  |  | Df | SumsOfSqs | MeanSqs | F.Model | R^2^ | Pr(>F) |
| --- | --- | --- | --- | --- | --- | --- | --- |
| C/N | C_N | 1 | 0.23949 | 0.23949 | 0.8683 | 0.08799 | 0.509 |
|  | Residuals | 9 | 2.48232 | 0.27581 | 0.91201 |  |  |
|  | Total | 10 | 2.7218 | 1 |  |  |  |

- 1. *Fraxinus rhynchophylla*

|  |  | Df | SumsOfSqs | MeanSqs | F.Model | R^2^ | Pr(>F) |
| --- | --- | --- | --- | --- | --- | --- | --- |
| Season | Season | 3 | 0.62425 | 0.20808 | 0.77037 | 0.22414 | 0.88 |
|  | Residuals | 8 | 2.16087 | 0.27011 | 0.77586 |  |  |
|  | Total | 11 | 2.78512 | 1 |  |  |  |

|  |  | Df | SumsOfSqs | MeanSqs | F.Model | R^2^ | Pr(>F) |
| --- | --- | --- | --- | --- | --- | --- | --- |
| pH | pH | 1 | 0.41584 | 0.41584 | 1.7551 | **0.14931** | 0.03 |
|  | Residuals | 10 | 2.36927 | 0.23693 | 0.85069 |  |  |
|  | Total | 11 | 2.78512 | 1 |  |  |  |

|  |  | Df | SumsOfSqs | MeanSqs | F.Model | R^2^ | Pr(>F) |
| --- | --- | --- | --- | --- | --- | --- | --- |
| TOC | TOC | 1 | 0.39768 | 0.39768 | 1.6657 | **0.14279** | 0.036 |
|  | Residuals | 10 | 2.38743 | 0.23874 | 0.85721 |  |  |
|  | Total | 11 | 2.78512 | 1 |  |  |  |

|  |  | Df | SumsOfSqs | MeanSqs | F.Model | R^2^ | Pr(>F) |
| --- | --- | --- | --- | --- | --- | --- | --- |
| TN | TN | 1 | 0.20784 | 0.20784 | 0.80642 | 0.07462 | 0.73 |
|  | Residuals | 10 | 2.57728 | 0.25773 | 0.92538 |  |  |
|  | Total | 11 | 2.78512 | 1 |  |  |  |

|  |  | Df | SumsOfSqs | MeanSqs | F.Model | R^2^ | Pr(>F) |
| --- | --- | --- | --- | --- | --- | --- | --- |
| NH_4_^+^ | NH4 | 1 | 0.33839 | 0.33839 | 1.383 | 0.1215 | 0.098 |
|  | Residuals | 10 | 2.44672 | 0.24467 | 0.8785 |  |  |
|  | Total | 11 | 2.78512 | 1 |  |  |  |

|  |  | Df | SumsOfSqs | MeanSqs | F.Model | R^2^ | Pr(>F) |
| --- | --- | --- | --- | --- | --- | --- | --- |
| TP | TP | 1 | 0.23196 | 0.23197 | 0.90854 | 0.08329 | 0.558 |
|  | Residuals | 10 | 2.55315 | 0.25532 | 0.91671 |  |  |
|  | Total | 11 | 2.78512 | 1 |  |  |  |

|  |  | Df | SumsOfSqs | MeanSqs | F.Model | R^2^ | Pr(>F) |
| --- | --- | --- | --- | --- | --- | --- | --- |
| Water | Water | 1 | 0.47495 | 0.47495 | 2.0559 | **0.17053** | 0.012 |
|  | Residuals | 10 | 2.31017 | 0.23102 | 0.82947 |  |  |
|  | Total | 11 | 2.78512 | 1 |  |  |  |

|  |  | Df | SumsOfSqs | MeanSqs | F.Model | R^2^ | Pr(>F) |
| --- | --- | --- | --- | --- | --- | --- | --- |
| TK | TK | 1 | 0.43559 | 0.43559 | 1.854 | **0.1564** | 0.021 |
|  | Residuals | 10 | 2.34952 | 0.23495 | 0.8436 |  |  |
|  | Total | 11 | 2.78512 | 1 |  |  |  |

|  |  | Df | SumsOfSqs | MeanSqs | F.Model | R^2^ | Pr(>F) |
| --- | --- | --- | --- | --- | --- | --- | --- |
| C/N | C_N | 1 | 0.40324 | 0.40324 | 1.693 | **0.14479** | 0.021 |
|  | Residuals | 10 | 2.38187 | 0.23819 | 0.85521 |  |  |
|  | Total | 11 | 2.78512 | 1 |  |  |  |
